# A151 THRESHOLD TRANSFERABILITY STUDY FOR 3 QUANTITATIVE FITS IN AN ACTIVE COLORECTAL CANCER SCREENING PROGRAM (CRCSP)

**DOI:** 10.1093/jcag/gwad061.151

**Published:** 2024-02-14

**Authors:** J Dube, R plantefeve, C Menard, M Bonin, J Rousseau, C Francois, A Caku

**Affiliations:** Biochemistry, Universite de Sherbrooke, Sherbrooke, QC, Canada; Hopital Fleurimont, Sherbrooke, QC, Canada; Biochemistry, Universite de Sherbrooke, Sherbrooke, QC, Canada; Biochemistry, Universite de Sherbrooke, Sherbrooke, QC, Canada; Biochemistry, Universite de Sherbrooke, Sherbrooke, QC, Canada; Biochemistry, Universite de Sherbrooke, Sherbrooke, QC, Canada; Biochemistry, Universite de Sherbrooke, Sherbrooke, QC, Canada

## Abstract

**Background:**

Three quantitative fecal immunochemical tests (FITs) were evaluated in the context of a possible supplier transition of the Quebec CRCSP. FITs from different suppliers have different sensitivities, specificities and positivity rates at preset thresholds (PT) which impacts the number of colonoscopies generated. Our hypothesis was that the determination of an equivalent threshold (ET) by a positivity rate equivalent strategy (PRES) or with regression analysis would lead to similar clinical performances.

**Aims:**

Determine if ETs can be obtained and what their impact is on the sensitivity and specificity of each supplier

**Methods:**

Kits containing a collection tube for each supplier were distributed to 6600 patients. Sampling was done on a fresh stool by patients with specific instructions for each supplier. Patients with FIT results ≥35 ug/g of stool hemoglobin were considered positive and referred for a colonoscopy within the CRCSP and those between 20 and 35 ug/g were invited to consent to a colonoscopy. Colonoscopy results were considered positive if advanced neoplasia (AN) was found. ETs were determined by PRES and regression analysis comparing each pair of suppliers. Median sensitivity and specificities were computed for each supplier at current and ETs.

**Results:**

Kits from 5513 patients were included in the study. Colonoscopy results were obtained for 262/342 FIT positive patients and 65/155 patients with results between 20 and 35 ug/g who consented to a colonoscopy. 69 patients were positive for AN at colonoscopy. ETs were obtained for the PRES but not for the regression method due to failed linearity tests. The ETs yielding the same positivity rate as Eiken’s 35.0 ug/g were determined to be 21.6 ug/g and 37.4 ug/g for Sentinel and Alfresa respectively. At the PT, the sensitivities of Alfresa (0.71, CI :0.58-0.80) and Eiken (0.71, CI:0.58-0.80) did not differ significantly but were higher than Sentinel (0.52, CI:0,39-0,64, p= 0.0059 and 0.016). The specificity for Sentinel (0.64, CI: 0.57-0.69) was significantly higher than Alfresa (0.45, CI: 0.38-0.50, p= 3.3E-6) and Eiken (0.54, CI: 0.47-0.59, p= 0.0059), as was the one from Eiken when compared to Alfresa (p =0.038). After adjustment using PRES, no significant differences for sensitivity (Alfresa (0.68, CI:0.55-0.77), Eiken (0.71, CI :0.58-0.80), Sentinel (0.52, CI:0.46-0.70)) and specificity (Alfresa (0.51 CI: 0.45-0.57), Eiken (0.54, CI: 0.47-0.59), Sentinel (0.57, CI: 0.50-0.62)) were observed between suppliers.

**Conclusions:**

Our study demonstrates that a PRES can be used to adjust the threshold in a supplier transition in order to achieve similar clinical performances. To our knowledge this is the first study to evaluate this in a real life active CRCSP setting where the stool is sampled by the patient and non-homogenized.

**Funding Agencies:**

Ministère de la Santé et des Services Sociaux du Québec and private funds (Somagen-Eiken, Quidel-Sentinel, Abbott-Alfresa)

